# The Comparative Efficacy of Burs Versus Piezoelectric Techniques in Third Molar Surgery: A Systematic Review Following the PRISMA Guidelines

**DOI:** 10.3390/medicina60122049

**Published:** 2024-12-12

**Authors:** Rocco Franco, Mattia Di Girolamo, Carlo Franceschini, Sofia Rastelli, Mario Capogreco, Maurizio D’Amario

**Affiliations:** Department of Life, Health and Environmental Sciences, School of Dentistry, University of L’Aquila, 67100 L’Aquila, Italy; rocco.franco@student.univaq.it (R.F.); mattia.digirolamo@student.univaq.it (M.D.G.); carlo.franceschini1@student.univaq.it (C.F.); mario.capogreco@univaq.it (M.C.); maurizio.damario@univaq.it (M.D.)

**Keywords:** third molar surgery, piezoelectric surgery, bur technique, postoperative pain, nerve damage, healing time, PRISMA

## Abstract

*Background and Objectives*: Third molar (wisdom tooth) extraction is one of the most common surgical procedures in oral and maxillofacial surgery. Traditional rotary instruments and burs have long been the standard tools for this procedure. However, recent advancements in surgical techniques, such as piezoelectric surgery, have gained popularity due to their purported advantages in terms of precision, safety, and postoperative outcomes. This systematic review aims to evaluate the efficacy, safety, and clinical outcomes of third molar surgery performed using burs versus piezoelectric surgery. *Materials and Methods*: This systematic review was conducted following the Preferred Reporting Items for Systematic Reviews and Meta-Analyses (PRISMA) guidelines. A comprehensive literature search was performed using the PubMed, Scopus, Web of Science, and Cochrane databases to identify relevant studies published up until October 2024. Randomized controlled trials (RCTs), clinical trials, and comparative studies assessing third molar surgery using either burs or piezoelectric instruments were included. The primary outcomes evaluated were surgical time, postoperative pain, swelling, nerve damage, and healing time. The data extraction and quality assessment were performed independently by two reviewers using standardized tools, and any discrepancies were resolved by a third reviewer. *Results*: A total of five studies met the inclusion criteria, and the meta-analysis revealed that piezoelectric surgery resulted in significantly lower postoperative pain and swelling compared to traditional bur techniques (*p* < 0.05). Additionally, the incidence of nerve injury was lower in the piezoelectric group, though the difference was not statistically significant. Surgical time was found to be longer with piezoelectric devices, but this was offset by improved healing outcomes and patient comfort. *Conclusions*: Piezoelectric surgery offers a less traumatic alternative to traditional burs for third molar extraction, with reduced postoperative morbidity and enhanced patient outcomes. Although the longer surgical duration may be a drawback, the overall benefits, particularly in terms of pain management and tissue preservation, support the adoption of piezoelectric techniques in clinical practice. Further high-quality randomized trials are recommended to strengthen the evidence base for these findings.

## 1. Introduction

Third molar surgery, commonly referred to as wisdom tooth extraction, remains one of the most frequently performed procedures in the field of oral and maxillofacial surgery. The removal of impacted or malpositioned third molars is often necessary to prevent or address complications such as pericoronitis, cyst formation, root resorption of the adjacent teeth, or crowding, particularly in orthodontic patients. While the need for this procedure is well established in clinical practice, the method using which third molars are extracted has evolved significantly over time, particularly with the introduction of new surgical techniques. Traditionally, burs powered by rotary instruments have been the primary tools utilized by clinicians. However, in recent years, piezoelectric surgery has emerged as a novel alternative, offering potential advantages in terms of precision, safety, and postoperative recovery [1,2].

The conventional method using rotary burs has been a reliable and efficient technique for decades, largely due to the ease with which it allows surgeons to section the teeth and remove the surrounding bone in order to facilitate tooth extraction. The high rotational speed and cutting efficiency of burs provide significant mechanical power, ensuring that even the most challenging extractions can be completed in a reasonable amount of time. Despite these advantages, the use of burs also carries inherent risks, particularly due to the excessive heat generated during bone cutting, which can damage the surrounding tissues; the risk of heat exposure can be attributed to the use of a tungsten carbide bur on a straight handpiece at 35,000 rpm. The use of angled handpieces with an excellent irrigation system makes it possible to eliminate the risk of overheating [3]. Additionally, the rotational movement of the burs can lead to inadvertent trauma to the adjacent soft tissues and structures, including the inferior alveolar nerve (IAN) and surrounding blood vessels. As a result, postoperative complications such as nerve injury, excessive bleeding, swelling, pain, and delayed healing have been associated with traditional bur-based third molar surgery [4].

Piezoelectric surgery, first introduced in the late 20th century, represents a technological innovation in oral surgery that seeks to address many of the challenges associated with the traditional rotary instruments. This technique employs ultrasonic oscillations to perform precise bone cutting while minimizing the trauma to the surrounding soft tissues [5]. The unique aspect of piezoelectric surgery lies in its selective cutting action, which targets mineralized tissues (i.e., bone) while sparing non-mineralized structures such as nerves, vessels, and mucosa. This selective tissue targeting offers significant safety benefits, particularly in procedures where the third molars are in close proximity to vital anatomical structures like the inferior alveolar nerve or the maxillary sinus. Furthermore, the reduction in heat generation associated with piezoelectric devices reduces the risk of thermal necrosis, a concern that is more pronounced for tungsten carbide burs used on a straight handpiece at 35,000 rpm [6,7,8,9].

One of the primary advantages of piezoelectric surgery that has gained attention in the literature is its potential to enhance postoperative recovery. Numerous studies have suggested that patients undergoing piezoelectric third molar surgery experience less postoperative pain, swelling, and trismus compared to those treated with burs [10]. This is attributed to the more conservative and less traumatic nature of the bone cutting performed when using piezoelectric devices. Additionally, because piezoelectric surgery is associated with reduced intraoperative bleeding due to its hemostatic effect on the soft tissues, the surgical field tends to be clearer, allowing for better visibility and precision during the procedure. However, the increased surgical time associated with piezoelectric surgery has been reported as a potential drawback, as the ultrasonic oscillations used in these devices tend to remove bone at a slower rate compared to the high-speed action of burs [11,12].

The comparative efficacy of piezoelectric and bur techniques in third molar surgery has become a topic of growing interest among oral surgeons, with several studies exploring the relative benefits and limitations of each approach. A systematic review is warranted to synthesize the available evidence and provide a comprehensive evaluation of both techniques. This review seeks to address critical questions, including whether the purported benefits of piezoelectric surgery—such as reduced postoperative pain, lower incidence of nerve injury, and improved soft tissue preservation—are consistently supported by the clinical data. Furthermore, we aim to assess whether the slower surgical times reported in piezoelectric surgery significantly affect the overall patient outcomes, particularly in terms of postoperative healing and recovery [13,14,15,16,17].

In the context of clinical decision-making, it is essential to evaluate the trade-offs between the efficiency of traditional burs and the tissue preservation benefits of piezoelectric surgery. While the primary goal of third molar surgery is to remove the impacted tooth with minimal complications, secondary considerations such as patient comfort, healing time, and long-term outcomes also play an important role in determining the optimal surgical technique. For patients at higher risk of complications, such as those with deeply impacted third molars close to the inferior alveolar nerve, piezoelectric surgery may offer a safer and more conservative approach. However, for routine cases where surgical efficiency is prioritized, traditional bur techniques may still be the preferred option due to their speed and familiarity among clinicians. Obviously, the two techniques of both classical and piezoelectric surgery must be interfaced in cases of surgery, especially of the lower wisdom teeth. In fact, with the piezoelectric insert alone, it is not possible to perform adequate odontotomy, while with the regular drills, it is possible to perform fast and effective odontotomy. Clinicians need to know the two techniques and how to apply them. In the case of odontotomy, the piezoelectric insert can be used to separate the crown in the section near the nerve structures [18,19,20,21,22,23,24].

Despite the increasing use of piezoelectric surgery in third molar extraction, a clear consensus on the superiority of one technique over the other has yet to be established. The studies available have reported mixed results, with some demonstrating clear advantages of piezoelectric surgery in terms of the postoperative outcomes, while others have highlighted the longer surgical duration as a potential limitation. Additionally, the cost of piezoelectric devices and the learning curve associated with their use may present barriers to their widespread adoption, particularly in resource-limited settings where traditional burs remain the standard of care.

The purpose of this systematic literature review with a meta-analysis is to evaluate the effectiveness of the classical and piezoelectric methods for use in wisdom tooth extraction surgery.

## 2. Materials and Methods

### 2.1. Eligibility Criteria

Documents were evaluated for their eligibility based on the Population, Exposure, Comparator, and Outcomes (PECO) framework:

Population (P): The participants included patients.

Exposure (E): Patients treated with piezoelectric surgery during third molar extraction, with difficulty assessed using the Pell and Gregory classification.

Comparator (C): Patients undergoing traditional surgical techniques for third molar extraction.

Outcome (O): The primary outcome was to compare the operative discomfort between the two surgical methods, assessed through VAS scores, swelling, trismus, and operative time. The secondary outcome was to analyze, via a meta-analysis, the difference in postoperative swelling between the two groups.

The inclusion criteria for the meta-analysis were as follows:

- Randomized clinical trials (RCTs);
- Patients requiring surgical extraction of third molars;
- Cases involving contralateral wisdom teeth with similar difficulty;
- Studies evaluating postoperative swelling;
- Articles published in English.

Exclusion criteria included the following:

- Studies in languages other than English;
- The full text being unavailable (e.g., posters or conference abstracts);
- Animal studies;
- Review articles;
- Case reports;
- The lack of an effective statistical analysis;
- The participants being heavy smokers (of more than 10 cigarettes per day);
- Significant medical history.
- Search Strategy

**Figure 1:**
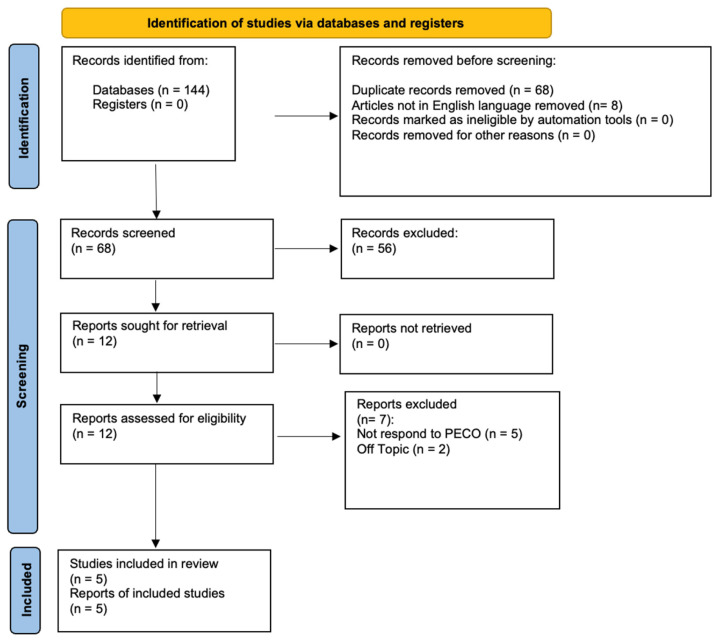
PRISMA flowchart.

A comprehensive literature search was conducted in the PubMed, LILACS, and Web of Science databases, covering articles published between 2000 and 31 December 2023. The search utilized the keywords “third molar” and “piezoelectric surgery” connected with the Boolean operator AND. MESH (Medical Subject Headings) terms were employed to refine the search strategy (Table 1). The review criteria are detailed in the PRISMA guidelines, with the search process outlined in the flowchart (Figure 1). Additionally, a manual review of the references from previous systematic reviews on related topics was performed.

This systematic review adhered to the PRISMA guidelines and the Cochrane Handbook for Systematic Reviews of Interventions. The review protocol was registered with the International Prospective Register of Systematic Reviews (PROSPERO), under registration number CRD42023396939.

### 2.2. Data Extraction

Two independent reviewers extracted data from the eligible studies using a customized Microsoft Excel template. Discrepancies were resolved through discussion or by consulting a third reviewer. The extracted data included the following:

(1) First author;
(2) Year of publication;
(3) Sample size;
(4) Study design;
(5) Parameters assessed;
(6) Methods for evaluating swelling;
(7) Results;
(8) Title;
(9) Summary;
(10) Effect of the intervention.

### 2.3. Quality Assessment

The quality of the studies included was evaluated using Version 2 of the Cochrane Risk of Bias tool for randomized trials (RoB 2). Any disagreements between the reviewers were resolved through discussion or consultation with a third reviewer.

### 2.4. Statistical Analysis

Data synthesis was performed using Review Manager software (version 5.2.8, Cochrane Collaboration, Copenhagen, Denmark, 2014). This study compared piezoelectric surgery and traditional surgery in terms of the VAS scores and postoperative swelling. The mean differences between the groups were calculated. Heterogeneity was assessed using the Higgins Index (I^2^) and the chi-square test, with the following thresholds: low (<30%), moderate (30–60%), and high (>60%) heterogeneity.

### 2.5. Evidence Grading

The Grading of Recommendations Assessment, Development, and Evaluation (GRADE) system was employed to assess the quality of the evidence and the level of certainty of the findings [25].

## 3. Results

### 3.1. Study Characteristics

At the end of the research, 144 studies were identified from the search conducted on the three engines. During the initial phase, 68 items were excluded because they were duplicates and 8 because they were not in English. Specifically, four articles were excluded from PubMed, one from Web of Science, and three from LILACS. During the initial screening phase, 56 articles were excluded from all search engines because they were systematic reviews of the literature and therefore did not meet the inclusion criteria; in addition, the filter of only randomized clinical trials being considered was included. During the final screening phase, the abstracts and the full text of 12 articles were evaluated. Only five were chosen in drawing up the present systematic study, as illustrated by the PRISMA 2020 flowchart in Figure 1; seven articles were excluded, as five did not meet the PECO framework principles, and two were off-topic. The remaining articles were selected for title and abstract screening according to the PECO model. Finally, these five articles were included in this publication on the search engines used. The studies considered have a time frame from 2011 to 2014. The studies analyzed were conducted in various parts of the world: Taiwan and Italy. A total of 208 subjects were analyzed.

All of the studies provided recruited impacted molars, and the majority used a split-mouth design. Both teeth in the arches had to have the same difficulty. The radiographic assessment was not uniform among the studies analyzed. In fact, some used the Pell and Gregory classification [26,27]; others had only inclusion as a criterion. All of the studies apart from Sivolella et al. were classified as split-mouth [28]. Sivolella performed surgery in the form of germectomies. All of the studies used piezoelectric surgery with the same modalities and frequencies. They also analyzed pain and discomfort using different classifications. Swelling was measured by taking zero-time fixed facial points. Piersanti’s study assessed postoperative discomfort using the Postoperative Symptom Severity (PoSSe) scale [29]. Pain was assessed using the VAS while swelling was assessed using the Schultze-Mosgau scale in Piersanti’s and Mantovani’s studies [27,29]. All of the studies evaluated pain with the VAS (Table 2 and Table 3).

### 3.2. Main Findings

Chang et al. conducted a randomized trial. This randomized crossover clinical trial comprised 20 patients with bilaterally, symmetrically impacted mandibular or maxillary third molars at the same level (18 women and 2 males, ages 17–29). The 40 impacted third molars were separated into two groups: the experimental group (n = 20), which underwent piezoelectric surgery utilizing a high-speed handpiece and a piezotome, and the control group (n = 20), which underwent traditional surgery using a high-speed handpiece and an elevator. An anonymous survey was used to evaluate the clinical parameters. The noise levels of the high-speed handpiece and the piezotome were measured and contrasted between the experimental and control groups. The individuals in the experimental group were less anxious about having their teeth pulled and had less face swelling and a lower force delivery during extraction than the patients in the control group. However, there were no discernible differences between the control and experimental groups in terms of noise-related disturbance, extraction time, amount of facial swelling, pain score, pain duration, or any noise levels produced by the devices under various conditions during tooth extraction. The VAS scores were evaluated after six days, and there were no significant statistical differences [30].

The study by Mantovani et al. evaluated the surgical differences between the two methods.

A single-center, randomized, split-mouth investigation was carried out on a group of unrelated healthy patients who had undergone surgical removal of both of their mandibular third molars at the University of Turin. At the same appointment, each patient received care that involved removal using burs on one side of the mandible and the use of a piezoelectric device on the opposite side. Postoperative pain, objective orofacial edema, and the length of the procedure were the main outcomes recorded; sex, age, and potential adverse events were the secondary outcomes. According to each treatment subgroup’s baseline differences, an ANOVA or a paired *t*-test was performed as appropriate, and a *t*-test was utilized to analyze the categorical data. A total of 100 patients who were otherwise healthy made up the study sample. Following 4 days, a statistical difference between the mean pain evaluation recorded by the patients who had undergone piezoelectric surgery and that experienced after bur (traditional) removal was reached (*p* = 0.043). However, the value used in the meta-analysis was that of the SEA collected on day 6. When compared to baseline, the clinical value of orofacial edema at day seven was reduced in the piezoelectric surgery group (*p* = 0.005).

In comparison to the piezoelectric surgery group, the average surgery time was considerably shorter in the bur group (*p* = 0.05).

Short-term problems (two dry sockets and one case of acute paresthesia) occurred in three individuals who underwent bur removal; all of them fully recovered after 4 weeks [27].

The study by Piersanti et Al. was designed as a split-mouth, randomized, unblinded clinical trial, and both molars had to have identical extraction difficulty scores. Piezoelectric surgery was used for extraction on the test side, while a traditional handpiece was used on the control side. The Postoperative Symptom Severity (PoSSe) scale, which was given to each patient, was used to measure the patients’ suffering. The secondary endpoints included pain, trismus, edema, and an assessment of the length of the surgery. The results within the subjects were compared using a repeated-measures analysis of variance and a paired-sample *t*-test. Ten patients, with a mean age of 22.4 ± 2.3 (6 F and 4 M), were enrolled. When compared to the use of a typical rotating handpiece, piezoelectric surgery had a considerably lower overall PoSSe scale score (24.7 ± 10.3 vs. 36.0 ± 7.6; *t*-test = −4.27; *p* = 0.002). A VAS assessment of 10 units was completed by the patients every day for 6 days postoperatively. There was no significant difference between the groups (repeated-measures ANOVA = 0.433, *p* = 0.519).

Additionally, compared to the use of traditional rotating handpieces, piezoelectric surgery resulted in considerably decreased postoperative edema after 1 week (2.75 ± 0.23 cm vs. 3.1 ± 0.39 cm; *t*-test = −2.63; *p* = 0.027) [29].

The study by Rullo was a prospective randomized clinical trial. A total of 52 patients with bilaterally and symmetrically oriented impacted mandibular third molars underwent surgical treatment utilizing a burr (Group A) on one random side of the lower jaw and a piezoelectric device (Group B) on the other side in prospective, randomized, split-mouth research. A modified Parant scale was used to classify “simple extractions” and “complicated extractions” in terms of the surgical difficulty. Variations in the surgery times between the groups and a comparison of the postoperative pain evaluation ratings on the Visual Analog Scale from day 0 to day 6 post-surgery were the primary outcome measures. In order to compare the levels of bone tissue damage caused by the two distinct procedures, bone samples were collected during surgery. Rotatory instruments were employed in “difficult extractions”, and reduced pain evaluation and much quicker operation times were noted. Similar surgical times were recorded for both procedures in “simple extractions”, but pain was worse on the day of surgery when a burr was utilized. Only the rotatory group showed signs of bone heat osteonecrosis, while the piezoelectric group had the highest levels of alkaline phosphatase.

The study by Sivolella et al. evaluated these two surgical evaluation methods [26].

A bilateral mandibular third molar germectomy was carried out, with one side undergoing piezoelectric surgery (piezo group) and the opposite side undergoing rotatory osteotomy (rotatory group) through random selection. The length of the surgical operation served as the predictor variable. The postoperative parameters (i.e., mouth opening range, clinical appearance of soft tissues, exudate, abscesses, wound dehiscence, locoregional lymphadenopathy, pain on palpation at the extraction site, persistent edema) at 7 and 30 days after surgery were the outcome variables. The outcome variables included the suitability of the method used, bleeding, and the postoperative parameters. Using the Visual Analog Scale, the patients documented their subjective postoperative pain every day for seven days. For the statistical analysis, a stepwise logistic regression model with binary variables and the Wilcoxon rank-sum test were utilized. A total of 26 patients (mean age: 15.4 years and 1.29 months) participated in the current study. For the piezo group, considerably more time was required to complete the osteotomy and extraction (15.77 vs. 6.56 min) than that required in the rotatory group (11.77 vs. 6.24 min; *p* = 0.028). The VAS score reported between the two groups after six days were 0.801 SD in the piezoelectric surgery and 0.647 in the traditional surgery (*p* = 0.476). Regarding the other outcome factors taken into consideration, there were no statistically significant differences between the two techniques [28].

### 3.3. Meta-Analysis

Two different meta-analyses were conducted. The first considered all five studies taken into account in terms of their assessment of the VAS scores after 6 days between the two different methods. In the statistical analysis, we standardized the SEA, and the only common parameter among the studies was that at day 6. Therefore, in the statistical analysis, we took that value at day 6 as the reference.

**Figure 2:**
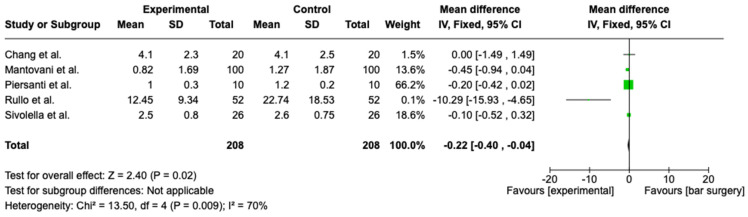
Forest plot evaluating VAS scores between two methods [26,27,28,29,30].

The meta-analysis was conducted according to a random model effect because of the high heterogeneity (I^2^ = 70%) among the five studies included that compared the VAS scores after six days. The overall effect, reported in a forest plot (Figure 2), revealed that the subjects operated on using piezoelectric surgery experienced less pain (Z = 2.40; *p* = 0.02), showing an association between piezoelectric surgery and VAS score.

**Figure 3:**
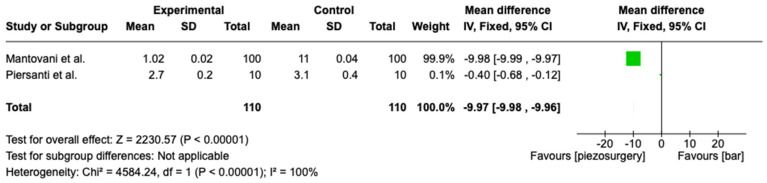
Forest plot evaluating swelling between two methods [27,29].

The second meta-analysis evaluated the swelling between bur surgery and piezoelectric surgery. This meta-analysis was conducted using a random model due to the heterogeneity (I^2^ = 100%). Two studies were included in this meta-analysis. The statistical analysis showed that the swelling was lower in the parts operated on using piezoelectric surgery (Z = 2230.17; *p* = 0.00001). This analysis showed that the swelling was lower in patients operated on using piezo surgery, with strong statistical evidence (Figure 3).

### 3.4. Bias Assessment

**Figure 4:**
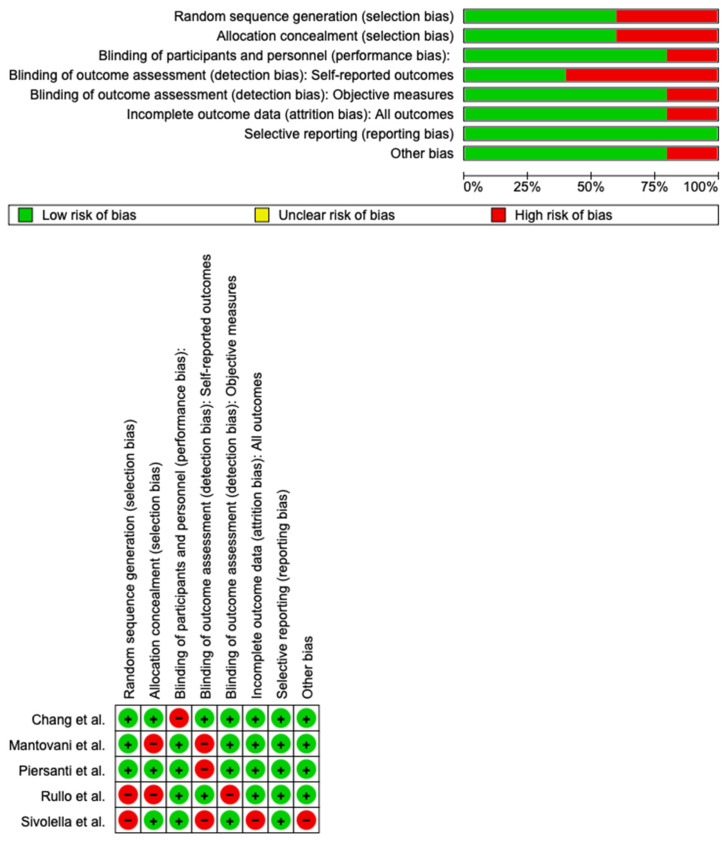
Bias assessment [26,27,28,29,30]. Green +: low risk of bias; red -: high rilsk of bias.

The risk of bias in the studies included is reported in Figure 4. The outcomes show a low risk of bias in all of the studies included. Table 4 evaluates the degree of evidence, and therefore we can say that the evidence for the results is high; however, there was heterogeneity in the studies.

## 4. Discussion

The comparative efficacy of the use of burs versus piezoelectric techniques in third molar surgery has been widely discussed in the literature due to the clinical implications for patient outcomes. Studies generally suggest that piezoelectric surgery, which utilizes ultrasonic oscillations to cut bone, offers certain advantages over the traditional bur technique, which uses rotary instruments. The most notable benefit of piezoelectric systems is their selective cutting ability, which minimizes damage to surrounding soft tissues like the nerves and blood vessels. This aspect is particularly important in lower third molar surgery, where the proximity to the inferior alveolar nerve can pose a significant risk of postoperative complications, such as nerve injury [31,32].

The comparison between piezoelectric surgery and conventional rotatory instruments for the surgical removal of third molars has become an area of significant interest due to their varying impacts on postoperative recovery and surgical outcomes [33,34]. Piezoelectric surgery, which utilizes ultrasonic micro-oscillations, offers several advantages, particularly in terms of its precision and tissue selectivity. This technique has been shown to selectively cut mineralized tissues like bone while sparing soft tissues such as the nerves and blood vessels, which is a critical benefit in surgeries that involve delicate anatomical areas. In contrast, conventional rotatory instruments, while they are effective and time-efficient, can generate substantial heat and mechanical trauma, potentially leading to complications like marginal osteonecrosis and delayed bone healing [26,27,35,36,37].

Studies employing a split-mouth randomized clinical trial design have demonstrated that patients treated with piezoelectric surgery experience less postoperative pain and swelling compared to those who undergo the procedure using conventional burs. Mantovani et al. observed that while the mean surgical time was longer with piezoelectric surgery, this group had significantly lower pain scores and reduced orofacial swelling at seven days postoperatively. The primary outcomes in favor of piezoelectric surgery included diminished pain (*p* = 0.043) and lower edema levels (*p* < 0.005), indicating that this technique’s micrometric precision and soft tissue preservation translate into a more favorable postoperative course. Similar findings were reported by Rullo et al., who noted that even in complex surgical scenarios, piezoelectric surgery produced less pain and soft tissue damage, contributing to faster recovery and a more predictable healing process [26].

The prolonged surgical duration associated with piezoelectric surgery is often cited as a major disadvantage. Multiple studies, including those by Sivolella et al. and Piersanti et al., have confirmed that piezoelectric surgery procedures took significantly longer to perform than conventional bur osteotomies [28,29]. For instance, Sivolella et al. reported that piezoelectric surgery required an average of 15.77 min for the procedure, compared to 11.77 min for conventional osteotomies (*p* = 0.028), highlighting the time-intensive nature of the technique [28]. This extended duration may contribute to greater intraoperative fatigue for surgeons and potentially influence patient satisfaction. However, the benefits observed in terms of postoperative outcomes, such as reduced trismus, swelling, and overall discomfort, may offset the longer procedure times in more complex extractions [29,30].

Another aspect to consider is the influence of piezoelectric surgery on bone healing and the preservation of structural integrity. The selective cutting ability of piezoelectric surgery reduces thermal and mechanical damage, thereby promoting a more favorable environment for bone regeneration. Histological evaluations have shown that piezoelectric surgery maintains a higher bone cell viability and minimizes the risk of osteonecrosis compared to conventional bur use, which can produce irregular bone surfaces and marginal necrosis due to high-temperature generation [26]. This may have long-term implications for procedures that require precise bone management, such as implant placements and other maxillofacial surgeries where bone healing quality is paramount. Patient-reported outcomes also tend to favor piezoelectric surgery. Piersanti et al. reported that patients treated with piezoelectric surgery rated their experience significantly higher on the Postoperative Symptom Severity (PoSSe) scale, which measures comfort, pain, and interference with daily activities. Patients undergoing piezoelectric surgery had lower scores, indicating reduced discomfort and fewer complications in their postoperative course [29]. Furthermore, Chang et al. found that patients preferred the reduced noise levels and vibration sensation associated with piezoelectric surgery, factors that contribute to an overall better surgical experience, especially in anxiety-prone individuals [30].

In conclusion, while the conventional rotatory technique remains faster and is thus suitable for routine third molar extractions, piezoelectric surgery offers substantial benefits in terms of soft tissue preservation, reduced postoperative morbidity, and enhanced patient comfort. The extended duration required for piezoelectric surgery may be a limitation, but its advantages in complex surgical cases and in situations where nerve preservation and reduced postoperative complications are crucial make it a valuable tool. Future studies focusing on optimizing piezoelectric surgery techniques to reduce the operative time without compromising its benefits could further solidify its role as a preferred method for third molar surgeries and other complex oral surgical procedures. Obviously, at the present time, it is not possible to use only one handpiece because some surgical steps, such as odontotomy, require a classical handpiece and a bur in order to allow for fast separation. In fact, it might be useful to investigate the creation of piezoelectric surgery inserts in order to decrease the instrumentation and increase the operator ergonomics. Our review also showed that patients operated on using piezo surgery experience benefits in terms of pain and swelling. Of course, only a few studies were taken into account, and in any case, we only considered two studies in the statistical analysis in Figure 3. In Figure 2, only the study by Rullo et al. gives significant results, which probably influences the outcome of the meta-analysis in favor of a lower VAS score in piezo surgery patients.

However, the bur technique remains a widely used method, particularly due to its familiarity among practitioners, faster bone removal, and cost-effectiveness. In some cases, the duration of surgery using piezoelectric systems is longer due to the slower bone-cutting process. This may be a limiting factor in situations where time is of the essence or when surgeons are more accustomed to the traditional tools.

## 5. Conclusions

In conclusion, while the conventional rotary instruments are faster and effective for routine third molar extractions, piezoelectric surgery offers significant advantages in terms of postoperative outcomes, particularly in reducing pain, swelling, and complications. The extended surgical duration associated with piezoelectric surgery can be seen as a limitation, but in cases where soft tissue preservation, nerve proximity, and patient comfort are critical, piezoelectric surgery provides a superior alternative. Future research should focus on optimizing the piezoelectric surgery techniques to reduce the operative time without compromising its benefits, making it an even more attractive option for oral and maxillofacial surgeons.

## Figures and Tables

**Table 1:** Search strategy.

PubMed (“third molar”) AND (“piezoelectric surgery”)
Web of Science ((ALL = (third molar)) AND ((ALL = (piezoelectric surgery))
LILACS “third molar” (palavras) AND “piezoelectric surgery” (palavras)

**Table 2:** Synthesis of data.

Author	Year	Sample	Design of the Study	Parameters Evaluated	Evaluation of Swelling	Results
Chang et al. [30]	2014	20 patients	Split-mouth, no scale for evaluation of the third molar. Bilaterally had to have the same difficulty.	Noise, degree of facial swelling, duration of pain	Results on swelling: Control: 6.25% no facial, 31.25% mild, 37.5% moderate, 25% severe; Study: 25% no facial, 25% mild, 40% moderate, 2% severe.	Less facial swelling than the patients in the control group
Mantovani et al. [27]	2014	100 patients	According to Pell and Gregory’s classification, split-mouth, only Class A or B and in position 1, 2, or 3. Bilaterally had to be similar.	Pain with the VAS; noise; swelling at 2, 7, 14, and 28 days	Results on swelling. Control: 1.10; Study: 1.02.	This study showed a statistically significant decrease in the group with piezoelectric surgery
Piersanti et al. [29]	2014	10 patients	Split-mouth, all teeth of equal difficulty and with inclusion that required a flap and osteotomy.	PoSSe scale, pain with the VAS, swelling after 7 days	Results on swelling: Control: 3.1 ± 0.39 cm; Study: 2.75 ± 0.23.	The clinical value for swelling was lower in the piezoelectric surgery group
Rullo et al. [26]	2012	52 patients	Split-mouth, symmetrically impacted third molars, evaluated with Pell and Gregory’s and Winter’s classifications.	The VAS, no evaluation of swelling		In the study groups, there was a shorter operational time and a lower VAS score.
Sivolella et al. [28]	2011	26 patients	Not blind, randomly assigned to two different treatments.	The VAS, clinical appearance of the soft tissue, swelling, operating time evaluated after 7 and 30 days	Results on swelling: Control: 30.8% of patients; Study: 26.9%	No statistical difference between the two methods

**Table 3:** Summary of the findings.

Authors	Title	Summary	Effects Between the Two Groups
Sivolella et al. [28]	Osteotomy for Lower Third Molar Germectomy: Randomized Prospective Crossover Clinical Study Comparing piezoelectric surgery and Conventional Rotatory Osteotomy	This study compared piezoelectric surgery and conventional rotatory osteotomy for mandibular third molar germectomy, finding that piezoelectric surgery took longer to complete but was otherwise comparable to the rotatory method in terms of the surgeon’s perception of its suitability and postoperative outcomes.	- The piezo-osteotomy took significantly longer to complete than the rotatory osteotomy (15.77 ± 6.56 min vs. 11.77 ± 6.24 min, *p* = 0.028). - There were no statistically significant differences between the two groups in terms of the following: - The surgeon’s perception of the suitability of the method; - Amount of intraoperative bleeding; - Postoperative outcomes, including mouth opening range, pain, and clinical signs/symptoms.
Rullo et al. [26]	Piezoelectric device vs. conventional rotative instruments in impacted third molar surgery: Relationships between surgical difficulty and postoperative pain with histological evaluations	This paper compared the use of a piezoelectric device to a conventional rotative instrument for the surgical removal of impacted third molars, evaluating the influence of surgical difficulty on postoperative pain and the histological differences between the two techniques.	Surgical time: - In complex extractions, the time needed for osteotomy and extraction was 28% longer with piezoelectric surgery compared to rotatory instruments (28.73 ± 5.46 min vs. 20.67 ± 4.46 min, *p* < 0.05). - In simple extractions, there was no significant difference in surgical time between the two techniques. Postoperative pain: - In simple extractions, pain on the day of surgery was significantly lower with piezoelectric surgery compared to rotatory instruments (*p* < 0.05). - In complex extractions, the pain from day 0 to day 6 was significantly higher with piezoelectric surgery compared to rotatory instruments (*p* < 0.05). Bone histology: - Piezoelectric surgery resulted in better preservation of the bone structure, with well-organized bone and no evidence of heat osteonecrosis. - The piezoelectric surgery samples had higher osteoblast activity and calcified nodule formation compared to the rotatory instrument samples.
Piersanti et al. [29]	Piezoelectric surgery or conventional rotatory instruments for inferior third molar extractions?	Piezoelectric surgery was more effective than traditional bur removal in reducing postoperative pain and swelling, though it took slightly longer on average to perform the surgery.	- Significantly lower postoperative discomfort as measured by the PoSSe scale (24.7 vs. 36.0, *p* = 0.002); - Significantly lower postoperative swelling at 1 week (2.75 cm vs. 3.1 cm, *p* = 0.027); - Significantly better scores in the speech, appearance, and interference with daily activity subscales of the PoSSe scale; - No significant differences in postoperative pain or trismus, except for less trismus on day 2 in the piezoelectric surgery group.
Mantovani et al. [27]	A split-mouth randomized clinical trial to evaluate the performance of piezoelectric surgery compared with traditional technique in lower wisdom tooth removal	Piezoelectric surgery was more effective than traditional bur removal in reducing postoperative pain and swelling, though it took slightly longer on average to perform the surgery.	- Postoperative pain: Piezoelectric surgery resulted in significantly lower pain scores compared to conventional bur removal, reaching statistical significance after 4 days (*p* = 0.043). - Orofacial swelling: Piezoelectric surgery resulted in significantly lower orofacial swelling at 7 days postoperatively compared to bur removal (*p* < 0.005). - Surgical duration: Conventional bur removal had a significantly shorter average surgical duration compared to piezoelectric surgery (*p* < 0.05). - Complications: Three patients in the bur removal group experienced short-term complications (two dry sockets, one case of temporary paresthesia), which resolved within 4 weeks. No complications were reported in the piezoelectric surgery group.
Chang et al. [30]	Comparison of clinical parameters and environmental noise levels between regular surgery and piezoelectric surgery for extraction of impacted third molars	This study found that piezoelectric surgery for the extraction of impacted third molars resulted in higher patient comfort, less soft tissue damage and facial swelling, and less pain and swelling in the initial days after the procedure compared to regular surgery.	- A total of 40% of piezoelectric surgery patients felt comfortable during the extraction vs. 25% of regular surgery patients (*p* < 0.05); - A total of 50% of piezoelectric surgery patients described the force as bearable vs. 20% of regular surgery patients (*p* < 0.05); - A total of 25% of piezoelectric surgery patients had no facial swelling vs. 6.25% of regular surgery patients (*p* < 0.01); - The piezoelectric surgery patients had less pain than the regular surgery patients on the operation day and the first day post-op.

**Table 4:** Grade assessment.

Certainty Assessment							No. of Patients		Effect		Certainty	Importance
No. of Studies	Study Design	Risk of Bias	Inconsistency	Indirectness	Imprecision	Other Considerations	[Intervention]	[Comparison]	Relative (95% CI)	Absolute (95% CI)		
New outcomes												
5	randomized trials	not serious	not serious	not serious	not serious	all plausible residual confounding would suggest a spurious effect, while no effect was observed.	110	110	-	see comment	⨁⨁⨁⨁ High	IMPORTANT

CI: confidence interval. ⨁: Quality of the included studies.

## Data Availability

All of the data are presented in this paper.
